# Variant allelic frequencies of driver mutations can identify gliomas with potentially false-negative *MGMT* promoter methylation results

**DOI:** 10.1186/s40478-023-01680-0

**Published:** 2023-11-02

**Authors:** Matthew McCord, Pouya Jamshidi, Vineeth Thirunavu, Lucas Santana-Santos, Erica Vormittag-Nocito, David Dittman, Stephanie Parker, Joseph Baczkowski, Lawrence Jennings, Jordain Walshon, Kathleen McCortney, Kristyn Galbraith, Hui Zhang, Rimas V. Lukas, Roger Stupp, Karan Dixit, Priya Kumthekar, Amy B. Heimberger, Matija Snuderl, Craig Horbinski

**Affiliations:** 1 Department of Pathology, Northwestern University Feinberg School of Medicine, Chicago, USA; 2 Department of Neurological Surgery, Northwestern University Feinberg School of Medicine, Chicago, USA; 3 Department of Pathology, New York University Langone Health, New York, USA; 4 Department of Preventive Medicine, Northwestern University Feinberg School of Medicine, Chicago, USA; 5 Department of Neurology, Northwestern University Feinberg School of Medicine, Chicago, USA; 6 Lou and Jean Malnati Brain Tumor Institute of the Robert H. Lurie Comprehensive Cancer Center, Northwestern University, Chicago, USA; 7 Feinberg School of Medicine, Northwestern University, 303 E Superior Street, 6-518, Chicago, IL 60611 USA

**Keywords:** Glioma, *MGMT* promoter, Temozolomide, Variant allelic frequency, Methylation

## Abstract

*MGMT* promoter methylation testing is required for prognosis and predicting temozolomide response in gliomas. Accurate results depend on sufficient tumor cellularity, but histologic estimates of cellularity are subjective. We sought to determine whether driver mutation variant allelic frequency (VAF) could serve as a more objective metric for cellularity and identify possible false-negative *MGMT* samples. Among 691 adult-type diffuse gliomas, *MGMT* promoter methylation was assessed by pyrosequencing (N = 445) or DNA methylation array (N = 246); VAFs of *TERT* and *IDH* driver mutations were assessed by next generation sequencing. *MGMT* results were analyzed in relation to VAF. By pyrosequencing, 56% of all gliomas with driver mutation VAF ≥ 0.325 had *MGMT* promoter methylation, versus only 37% with VAF < 0.325 (*p* < 0.0001). The mean *MGMT* promoter pyrosequencing score was 19.3% for samples with VAF VAF ≥ 0.325, versus 12.7% for samples with VAF < 0.325 (*p* < 0.0001). Optimal VAF cutoffs differed among glioma subtypes (IDH wildtype glioblastoma: 0.12–0.18, IDH mutant astrocytoma: ~0.33, IDH mutant and 1p/19q co-deleted oligodendroglioma: 0.3–0.4). Methylation array was more sensitive for *MGMT* promoter methylation at lower VAFs than pyrosequencing. Microscopic examination tended to overestimate tumor cellularity when VAF was low. Re-testing low-VAF cases with methylation array and droplet digital PCR (ddPCR) confirmed that a subset of them had originally been false-negative. We conclude that driver mutation VAF is a useful quality assurance metric when evaluating *MGMT* promoter methylation tests, as it can help identify possible false-negative cases.

**Supplementary Information:**

The online version contains supplementary material available at 10.1186/s40478-023-01680-0.

## Introduction

Temozolomide (TMZ) improves survival in high-grade gliomas [1–3] and is standard of care for these tumors [4]. TMZ alkylates DNA bases in tumor cells, especially guanine. O^6^-methylguainine is a common product of TMZ alkylation, and plays a key role in TMZ-driver tumor cell apoptosis. During DNA replication, O^6^-methylguanine residues erroneously pair with thymine on the new DNA strand. DNA mismatch repair enzymes excise the thymine residues, but methylguanine residues persist on the template DNA strand, triggering a series of “futile repair” cycles, leading to tumor cell apoptosis [5, 6]. The enzyme O^6^-methylguanine-DNA-methyltransferase (MGMT) counteracts TMZ by removing methyl groups from O^6^-methylguanine residues [7–9]. The gene encoding the enzyme, *MGMT*, is located at 10q26 [10], and methylation of its promoter inhibits gene expression, making tumor cells more vulnerable to TMZ [11–13] and improving clinical response to the drug [14]. *MGMT* promoter methylation is also associated with improved prognosis even in the absence of TMZ treatment [15].

Due to its importance in prognosis and in predicting TMZ response, testing for *MGMT* promoter methylation is required in the diagnostic workup of adult-type diffuse gliomas. Multiple approaches have been developed for testing *MGMT* promoter methylation, including pyrosequencing, methylation-specific PCR, methylation-specific high-resolution melting, and MethyLight™. Because pyrosequencing is simple, reproducible, and a good predictor of TMZ response, it has become the most widespread test [16–19]. More recently, genomic DNA methylation array and droplet digital PCR (ddPCR) have emerged as alternative methods [20–24].

In *MGMT* pyrosequencing, the promoter is usually considered “methylated” (positive) when ≥ 10% of measured CpG sites in the promoter sequence are methylated, and “unmethylated” (negative) when < 10% are methylated. This cutoff has been validated clinically [17] and is used for *MGMT* promoter testing at many institutions, although other cutoffs have been suggested [25, 26]. Accurate results depend on sufficient tumor cellularity in analyzed samples. Methylation of the promoter is abnormal, occurring only in neoplastic cells. Thus, if tumor cellularity is too low in a specimen, with too many admixed non-neoplastic cells (including astrocytes, neurons, inflammatory cells, etc.), *MGMT* promoter methylation signals could be diluted, leading to a false-negative result. A minimum of 70% tumor cellularity is preferred for *MGMT* promoter analysis, a cutoff also recommended in other diagnostic assays such as methylation profiling arrays [27, 28]. Tumor cellularity is usually estimated via subjective light microscopic evaluation of formalin fixed, paraffin embedded (FFPE) tumor tissue stained with hematoxylin and eosin (H&E). Moreover, specimens with < 70% estimated tumor cellularity are nearly always still tested if no better tissue block is available.

Adult-type diffuse gliomas usually have characteristic driver mutations whose relative amounts in an analyzed sample are quantifiable by next generation sequencing (NGS). *TERT* promoter mutation is found in ~ 85% of IDH-wildtype glioblastomas and > 95% of IDH-mutant and 1p/19q co-deleted oligodendrogliomas [29, 30]. Mutations in *IDH1* or *IDH2* are disease-defining features of IDH-mutant and 1p/19q co-deleted oligodendroglioma and IDH-mutant astrocytoma [31]. These specific driver mutations are almost always heterozygous and are not usually affected by copy number alterations [32–34]. Thus, tumor cellularity in most adult-type diffuse gliomas can be estimated by simply doubling the measured variant allelic frequency (VAF) of the driver mutation. Previous work, including our own, supports this approach for quantifying tumor cellularity [35, 36].

Here, we investigated driver mutation VAF as a way to evaluate *MGMT* promoter methylation test results by pyrosequencing, DNA methylation array, and ddPCR in a large single-institution observational study of adult-type diffuse gliomas as defined by the 5th edition of the WHO classification [31]. The central hypothesis was that glioma samples with low VAF are at risk of false negative *MGMT* assay results due to dilution of tumor methylation signals by non-neoplastic cells.

## Materials and methods

### Collection of patient samples

**Fig. 1:**
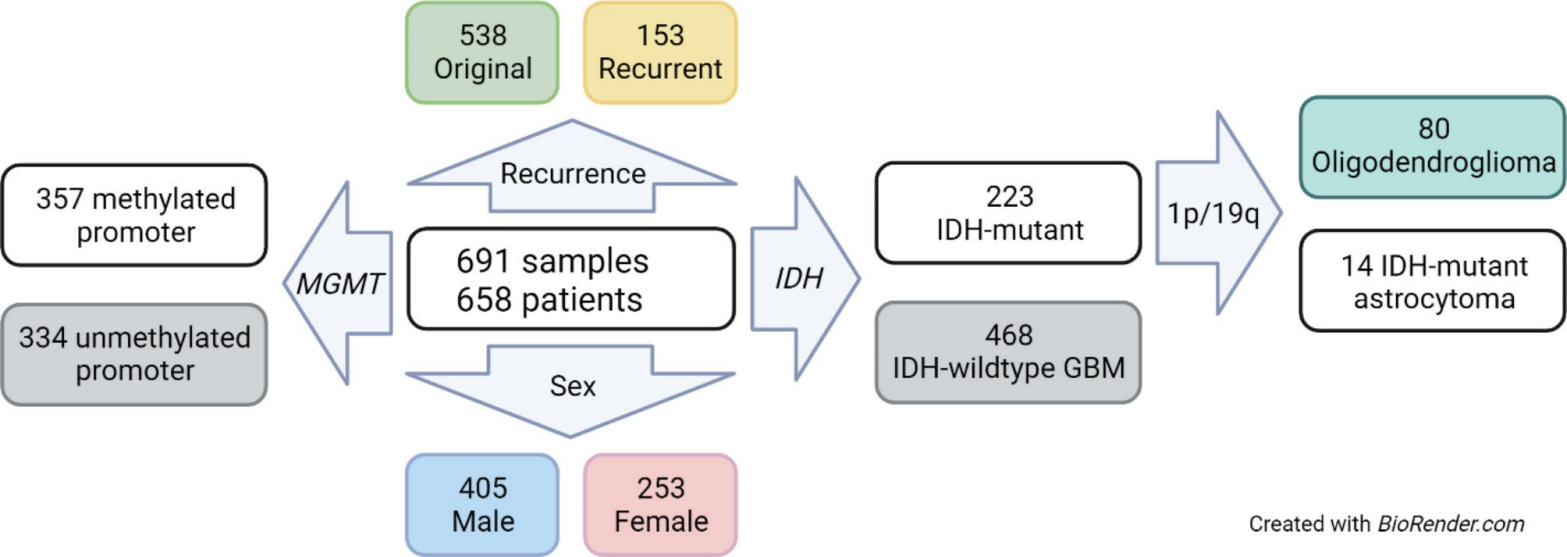
**Cohort characteristics**. Flowchart for key characteristics of tumor samples and patients. Illustration prepared with Biorender. GBM: IDH-wildtype glioblastoma

The cohort comprised 691 consecutive CNS WHO grade 2–4 adult-type diffuse gliomas from 658 patients resected between 2006 and 2022. Between 2019 and 2022, all tumors underwent NGS and *MGMT* promoter methylation testing, the latter by pyrosequencing (445 samples) or by DNA methylation array (246 samples) at Northwestern Memorial Hospital. All tumors were classified according to 2021 WHO guidelines [31]. Tumor types included glioblastoma, IDH-wildtype, CNS WHO grade 4 (IDHwt GBM); IDH-mutant astrocytoma, CNS WHO grades 2–4 (IDHmut astrocytoma); and IDH mutant and 1p/19q co-deleted oligodendroglioma CNS WHO grades 2–3 (IDHmut oligodendroglioma). Among IDHwt GBMs, only those with *TERT* promoter mutation were included due to the high frequency of that mutation in IDHwt GBM and its utility as a marker of tumor purity [35, 36]. *MGMT* and NGS data were obtained from the same tissue block in each case. Key characteristics of the patient cohort are summarized in Table 1. Characteristics of tumor samples and patients are also illustrated in Fig. 1.

**Table 1:** Patient cohort characteristics

Variable		IDHwt GBM	IDHmut astro	IDHmut oligo
Sex	Male	278	78	49
	Female	170	56	27
Age	median age (range)	64 (33–91)	37 (19–74)	44 (21–78)
Grade	CNS WHO grade 2	N/A	36	38
	CNS WHO grade 3	N/A	55	38
	CNS WHO grade 4	448	43	N/A
Original vs. recurrent	original tumor	391	90	54
	recurrent tumor	57	44	22
*MGMT* assay	*MGMT* promoter pyrosequencing assay	291	88	49
	*MGMT* promoter methylation array	157	46	27
*MGMT* promoter status	*MGMT* promoter methylated	172	94	73
	*MGMT* promoter unmethylated	276	40	3
Driver mutation VAF	Mean driver mutation VAF (range)	0.34 (0.02–0.84)	0.38 (0.02–0.79)	0.39 (0.07–0.51)
	Median driver mutation VAF	0.34	0.40	0.40

IDHwt GBM: IDH-wildtype glioblastoma; IDHmut astro: IDH-mutant astrocytoma; IDHmut oligo: IDH-mutant and 1p/19q co-deleted oligodendroglioma

### *MGMT* promoter methylation testing

All *MGMT* promoter methylation assessments were carried out according to manufacturer instructions for each respective assay. For pyrosequencing, DNA was extracted from FFPE tissue using Purigen Biosystems (Pleasanton, CA, USA), then bisulfite-modified using the Qiagen EpiTect bisulfite Kit (Qiagen). DNA was amplified by PCR, and pyrosequencing was performed using primers in four CpG sites in exon 1 of the human *MGMT* gene (sequence on chromosome 10 from 131,265,519 to 131,265,537: CGACGCCCGCAGGTCCTCG). PyroGold Q24 SQA Reagents and the Pyro Q-CpG software on a PyroMark ID pyrosequencer (Qiagen, Crawley, UK) were used. For DNA methylation array, analysis of two CpG sites in the *MGMT* promoter (cg12434587, chr10:131,265,209–131,265,210 and cg12981137, chr10:131,265,575–131,265,576) was performed according to the Bady algorithm as previously described [20]. Briefly, the M-values of the methylated and unmethylated intensities at those 2 positions were used as input in a logistic regression model (MGMT-STP27). The *MGMT* score was obtained by logit-transformation of the probability that the *MGMT* promoter is methylated. The predicted probabilities, *MGMT* score, confidence intervals, and *MGMT* classification were directly obtained by the function *MGMT* predict from the R package mgmtstp27 (https://github.com/badozor/mgmtstp27). *MGMT* assessment via droplet digital PCR (ddPCR) was performed as previously described [24]. Briefly, genomic DNA samples were subjected to bisulfite conversion using the Epi-Tect Fast Bisulfite Conversion kit as per manufacturer’s instruction (Qiagen, Germantown MD, USA). *MGMT* promoter methylation was quantified using primers and probes targeting the converted and unconverted template (Supplementary Table S1) and ddPCR Supmermix for Probes as per manufacturer’s instructions (Bio-Rad, Hercules, CA, USA). Our ddPCR assay targeted the same 2 *MGMT* promoter CpG sites as the Bady algorithm, which show good correlation with TMZ response and survival [37].

### Next generation sequencing

Two commercially available NGS panels were used. Oncomine Comprehensive Assay Version 3 (OCAV3) was performed on 519 cases between 2019 and 2022, according to manufacturer’s instructions. PGDx Solid Tumor NGS (adopted by Northwestern Memorial Hospital in 2022) was performed on 172 cases, according to the manufacturer’s recommendations. Detailed methodological descriptions for both NGS panels can be found in the “Supplementary Methods” section.

### TERT promoter droplet digital PCR

*TERT* promoter mutation assessment by droplet digital PCR (ddPCR) was performed using the BioRad™ QX200 ddPCR system, according to the manufacturer’s recommendations. Briefly, genomic DNA was extracted, and partitioned into droplets. The PCR reaction was performed using primers for the two most common *TERT* promoter mutations (c.-124 C > T and c.-146 C > T). Results were quantified and analyzed using a droplet reader, as previously described [38].

### DNA methylation array-based tumor classification

For each of the 246 samples in which the *MGMT* promoter was assessed by genomic DNA methylation array, DNA methylation-based tumor classification was also performed using our previously validated in-house classifier [21, 39]. Briefly, extracted DNA underwent bisulfite conversion using the EZ-96 DNA methylation kit (Zymo Research Corp., Tustin, CA). After denaturation with 0.1 N NaOH and cleaning with a ZR-96 DNA concentrator-5 kit (Zymo Research Corp.), bisulfite-converted DNA was hybridized to Infinium Human Methylation EPIC BeadChips (EPIC, 850 K). Arrays were scanned and IDAT files generated. Each BeadChip had quality metrics assessed for red and green staining, hybridization, and bisulfite conversion. Methylation array data was analyzed using our classifier. Cases with methylation class score ≥ 0.9 were considered a match.

### Data collection

For each tumor sample, pathology reports were reviewed. Nine cases needed their final diagnoses adjusted to fit with the 2021 WHO classification system based on molecular and morphologic criteria.

### Parallel testing from different tumor regions

We performed parallel testing for *TERT* promoter VAF and *MGMT* promoter methylation on 5 IDH-wildtype GBM samples from the Northwestern University Nervous System Tumor Bank (NSTB). Each sample had previously undergone pathology review and clinical molecular testing, had an established diagnosis of IDH-wildtype GBM with *TERT* mutation, and had a positive test for *MGMT* promoter methylation by DNA methylation array. For each sample, one area of at least 0.5 cm^2^ with the highest relative cellularity (estimated by light microscopy of an H&E section) and a corresponding area of similar size with the lowest relative cellularity were differentially delineated via light microscopy of H&E slides. *TERT* VAF and *MGMT* promoter methylation were measured in parallel by ddPCR on tissue dissected from each delineated area.

### Re-testing of tumor samples

We re-tested 12 IDHwt GBM samples (Table 2) for *MGMT* promoter methylation, by both DNA methylation array and ddPCR, that had previously been tested by pyrosequencing. Samples were selected based on tissue availability, *TERT* VAF, and availability of clinical follow-up data. In all cases, re-testing was performed on the same FFPE tissue blocks that had originally been tested with pyrosequencing. Six samples had *TERT* VAF < 0.1, and 6 had VAF > 0.25. Samples from the low-VAF and high-VAF cohorts were sex- and age-matched.

**Table 2:** Analysis of differentially dissected tumor samples

Sample	*TERT* promoter mutation	High cellularity area			Low cellularity area		
		Microscopic cellularity	*TERT* VAF	*MGMT* level, %	Microscopic cellularity	*TERT* VAF	*MGMT* level, %
1	c.-124 C > T	60%	0.311	52.213	10%	0.152	18.800
2	c.-124 C > T	80%	0.214	30.332	20%	0.077	4.553
3	c.-124 C > T	80%	0.376	59.323	5%	0.056	11.865
4	c.-124 C > T	60%	0.386	67.633	40%	0.440	75.486
5	c.-124 C > T	90%	0.315	68.293	50%	0.263	57.687

All MGMT ddPCR results were positive, in both high- and low-cellularity areas of tumors

### Data processing and statistical analysis

Differences between observed versus expected frequency of positive and negative test results were compared with Fisher’s exact test. The Fisher’s exact test function of Cutoff Finder [40], available at https://molpathoheidelberg.shinyapps.io/CutoffFinder_v1/, was used to identify VAF cutoff points with maximal differences in test outcomes. *TERT* mutation VAF was used for analysis of IDHwt GBM, and *IDH1/2* VAF for both IDHmut astrocytoma and IDHmut oligodendroglioma. Differences between means of 2 groups were compared with Student’s t-test. Non-parametric comparisons between 2 groups were done with Mann-Whitney *U* test (Wilcoxon rank-sum test).

To calculate cumulative mean positivity rates of *MGMT* promoter assays, samples were ranked by increasing VAF, and the mean positivity rate was measured for each sample plus all samples below it. This process was repeated successively (mean of the first 2 samples with the lowest VAF, then the first 3, etc.) until the entire cohort had been measured. Cumulative means and medians were calculated for *MGMT* promoter pyrosequencing scores in a similar fashion. Simple linear regression was performed comparing *MGMT* promoter pyrosequencing score to driver mutation VAF for each sample. Two-part linear regressions were performed on cumulative mean positivity rate data to analyze the variation in likelihood of positive *MGMT* pyrosequencing results with increasing driver mutation VAF. The data curves for cumulative mean positivity rate of each glioma subtype showed two distinct portions. Regression lines were fitted to both portions of each curve. In all cases, the slope of the line at lower VAF values was much steeper than at high VAF values. The regression lines were separated at the VAF value where the slopes had the greatest magnitude of difference (i.e., slope value *M* for the low-VAF regression line minus slope value *M* for the high-VAF regression line). All VAF values were evaluated for each pair of regression lines. All linear regressions are described in terms of slope values of best-fit lines, R^2^ values for goodness-of-fit, and *p*-values for slope deviation from zero. Tumor cellularity was estimated from *TERT* or *IDH* driver mutation VAF by simply doubling VAF values and multiplying by 100% (these mutations are usually heterozygous). In rare cases with VAF > 0.5, the cellularity estimate was capped at 100%.

For all statistical tests, *p*-values less than 0.05 were considered significant. For non-parametric tests, approximate *p*-values are reported. Data organization, processing, statistical analysis, and figure preparation were carried out with Microsoft Excel 2016, GraphPad Prism 5, Cutoff Finder, and BioRender.

## Results

### Cohort characteristics

The cohort was composed of 691 consecutive adult-type diffuse glioma samples, including 468 IDHwt GBMs, 144 IDHmut astrocytomas, and 80 IDHmut oligodendrogliomas. Tumor samples were from 658 patients (405 male, 253 female), 153 samples were from residual/recurrent tumors, and 36 were paired original and recurrent tumors. Among 445 samples assessed via pyrosequencing, 217 (49%) tested positive for *MGMT* promoter methylation (101 IDHwt GBMs, 68 IDHmut astrocytomas, 48 IDHmut oligodendrogliomas), while 228 (51%) tested negative (200 IDHwt GBMs, 25 IDHmut astrocytomas, 3 IDHmut oligodendrogliomas). Among 246 samples assessed via DNA methylation array, 140 (57%) tested positive for *MGMT* promoter methylation (79 IDHwt GBMs, 32 IDHmut astrocytomas, 29 IDHmut oligodendrogliomas), while 106 (43%) tested negative (88 IDHwt GBMs, 18 IDHmut astrocytomas, 0 IDHmut oligodendrogliomas). Among the 246 cases analyzed by methylation array, 194 (79%) had a methylation classifier score ≥ 0.9. One tumor matched to methylation class “control tissue: inflammatory tumor microenvironment,” but morphologic and molecular features indicated a diagnosis of IDH-wildtype GBM; NGS revealed very low *TERT* VAF (0.09) in that case. Despite the low VAF, the tumor tested positive for *MGMT* promoter methylation on the array. Among the 36 paired original-recurrent samples, recurrent tumors from two patients had discordant *MGMT* results with the primary tumors. In one case, a newly diagnosed grade 4 IDH-mutant astrocytoma was positive for *MGMT* promoter methylation on pyrosequencing (35.9% CpG site methylation), but the recurrent tumor 8 months later was negative on pyrosequencing (5.4%). In the second case, a newly diagnosed IDH-wildtype GBM was negative by pyrosequencing (3.9%), but the recurrence 4 months later was positive by methylation array. Patient characteristics are summarized in Table 1. Cohort characteristics are illustrated in Fig. 1. Additional details are available in Supplementary Table S2 (pyrosequencing data) and Supplementary Table S3 (DNA methylation array data). Details of the paired original and recurrent samples are available in Supplementary Table S4.

### *MGMT* promoter methylation outcomes by pyrosequencing according to driver mutation VAF in adult-type diffuse gliomas

**Fig. 2:**
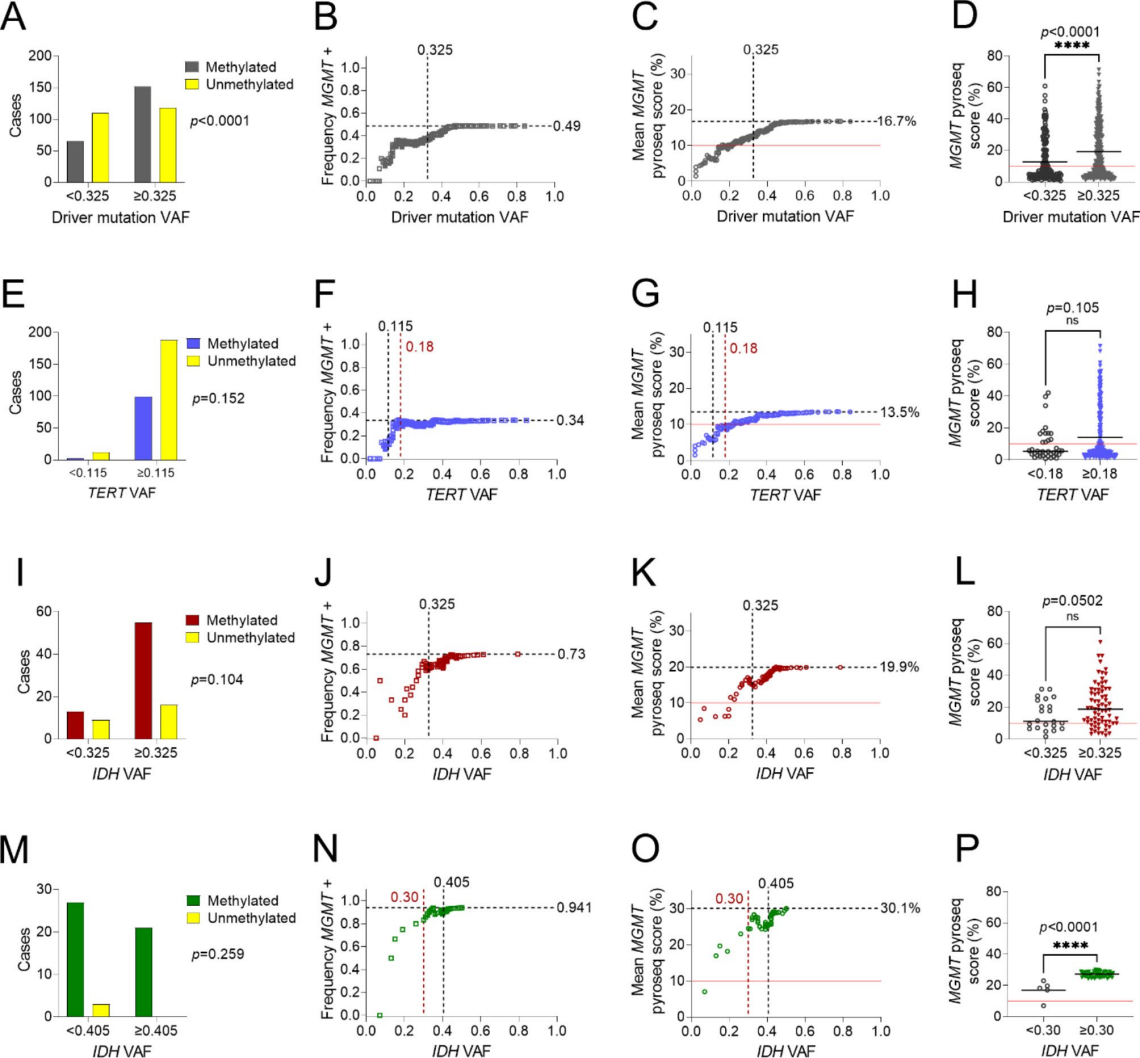
***MGMT*promoter methylation trends in pyrosequencing samples**. (**A**) Frequency of positive results for all glioma samples above and below the cutoff value of VAF = 0.325. (**B**) Cumulative mean frequency of positive test results as a function of VAF. (**C**) Trends in cumulative mean *MGMT* promoter pyrosequencing score with increasing VAF. (**D**) Mean *MGMT* promoter pyrosequencing scores above and below VAF = 0.325. Similar results are shown for each tumor subtype, including IDH-wildtype glioblastoma (**E**-**H**), IDH-mutant astrocytoma (**I**-**L**) and IDH-mutant and 1p/19q co-deleted oligodendroglioma (**M**-**P**). (Horizontal dashed black lines: mean values for cohort; Horizontal solid red lines: *MGMT* positivity cutoff of 10.0%; Vertical dashed black lines: cutoff values identified by Cutoff Finder; Vertical dashed red lines: cutoff values identified by multi-part linear regression; Panels A, E, I, M: Fisher’s exact test; Panels D, H, L, P: unpaired Student’s T-test; pyroseq: pyrosequencing; *p < 0.05; ****p < 0.0001)

Among all adult-type diffuse gliomas combined, the optimal driver mutation VAF (either *TERT* for IDHwt GBM or *IDH1/2* for IDHmut astrocytoma and IDHmut oligodendroglioma) in the context of *MGMT* promoter methylation pyrosequencing was determined by Cutoff Finder at 0.325 (Supplementary Figure S1A). At or above that cutoff, 152/270 samples (56%) tested positive for *MGMT* promoter methylation, compared to 65/175 (37%) below (*p* < 0.0001 via Fisher’s exact test, Fig. 2A). We next plotted cumulative mean *MGMT* promoter positivity rates as a function of driver mutation VAF. Results for all gliomas pooled together showed that maximal positivity rates were at a VAF of ~ 0.48 or higher, with intermediate positivity rates between 0.18 and 0.48. Below a VAF of 0.18, the likelihood of a given *MGMT* test being positive sharply dropped (Fig. 2B). We then plotted the cumulative mean *MGMT* promoter pyrosequencing score (percent of methylated CpG sites) as a function of driver VAF. The curve did not plateau until VAF was above 0.325 (Fig. 2C). Mean *MGMT* score at or above a VAF of 0.325 was 19.3% versus 12.7% below (*p* < 0.0001 via unpaired Student’s t-test, Fig. 2D). Mann-Whitney U test showed significant variation in median scores above (13.2%) and below (5.7%) this cutoff (*p* < 0.0001, Supplementary Figure S2A), keeping in mind that the standard cutoff for a “methylated” pyrosequencing result is 10%. The curve for cumulative median scores showed a sigmoid shape, with distinct plateaus above VAF = 0.06 and VAF = 0.4 (Supplementary Figure S2B).

Optimal VAF cutoffs via categorical Fisher’s exact tests in Cutoff Finder for the 3 major glioma subtypes (Supplementary Figure S1B-D) were 0.115 for IDHwt GBM (Fig. 2E), 0.325 for IDHmut astrocytoma (Fig. 2I), and 0.405 for IDHmut oligodendroglioma (Fig. 2M). While none of those exact cutoffs were statistically significant (see Supplementary Table S5 for details), there were clearly different slopes of *MGMT* promoter methylation positivity curves as a function of VAF within each glioma subtype (Fig. 2F J, and 2 N). For each curve, we performed multi-part linear regression analyses as an alternative approach to identifying cutoffs. We found that the slopes were maximally different at subtype-specific VAF inflection points: *TERT* VAF = 0.18 for IDHwt GBM, *IDH* VAF = 0.325 for IDHmut astrocytoma, and *IDH* VAF = 0.30 for IDHmut oligodendroglioma (Supplementary Figures S2C, S2F, and S2I, respectively). Cumulative mean *MGMT* pyrosequencing score trends are shown for IDHwt GBM (Fig. 2G), IDHmut astrocytoma (Fig. 2K), and IDHmut oligodendroglioma (Fig. 2O). Cumulative mean trends for each individual CpG pyrosequencing site are shown in Supplementary Figure S3. Mean *MGMT* scores for samples above and below VAF values identified by Cutoff Finder did not show statistically significant differences via unpaired student’s t-test (not shown). For IDHwt GBM, mean *MGMT* scores above and below the *TERT* VAF cutoff of 0.18 (identified by multi-part linear regression) were 14.06% (in the “methylated” range) and 9.56% (in the “unmethylated” range), respectively (*p* = 0.105 via unpaired student’s t-test, Fig. 2H). For IDHmut astrocytoma (Fig. 2L), Cutoff Finder and regression both identified the same VAF cutoff at which the difference in mean *MGMT* scores maximize (*IDH* VAF = 0.325, *p* = 0.05). For IDHmut oligodendroglioma (Fig. 2P), mean *MGMT* score differences above and below the regression cutoff (*IDH* VAF = 0.30) did reach statistical significance (*p* < 0.0001). Mann-Whitney *U* tests based on regression cutoffs for glioma subtypes showed significant variation of median *MGMT* promoter methylation scores only for IDHmut oligodendroglioma (18.2% vs. 27.2%, *p* = 0.0003, Supplementary Figure S2D, S2G, S2J). Cumulative median trends are shown in Supplementary Figures S2E, S2H, and S2K.

Together, these data suggest that, since the rate of *MGMT* promoter methylation drops sharply below a *TERT* promoter VAF of ~ 0.18 (i.e., ~ 35–40% tumor cellularity) for IDHwt GBM, cases with such low VAF may be at increased risk of false-negative *MGMT* promoter methylation results by pyrosequencing. Similar cutoffs are at ~ 0.33 and 0.30–0.40 for IDHmut astrocytomas and IDHmut oligodendrogliomas, respectively.

### *MGMT* promoter methylation test results by genomic DNA methylation array according to driver mutation VAF in adult-type diffuse gliomas

**Fig. 3:**
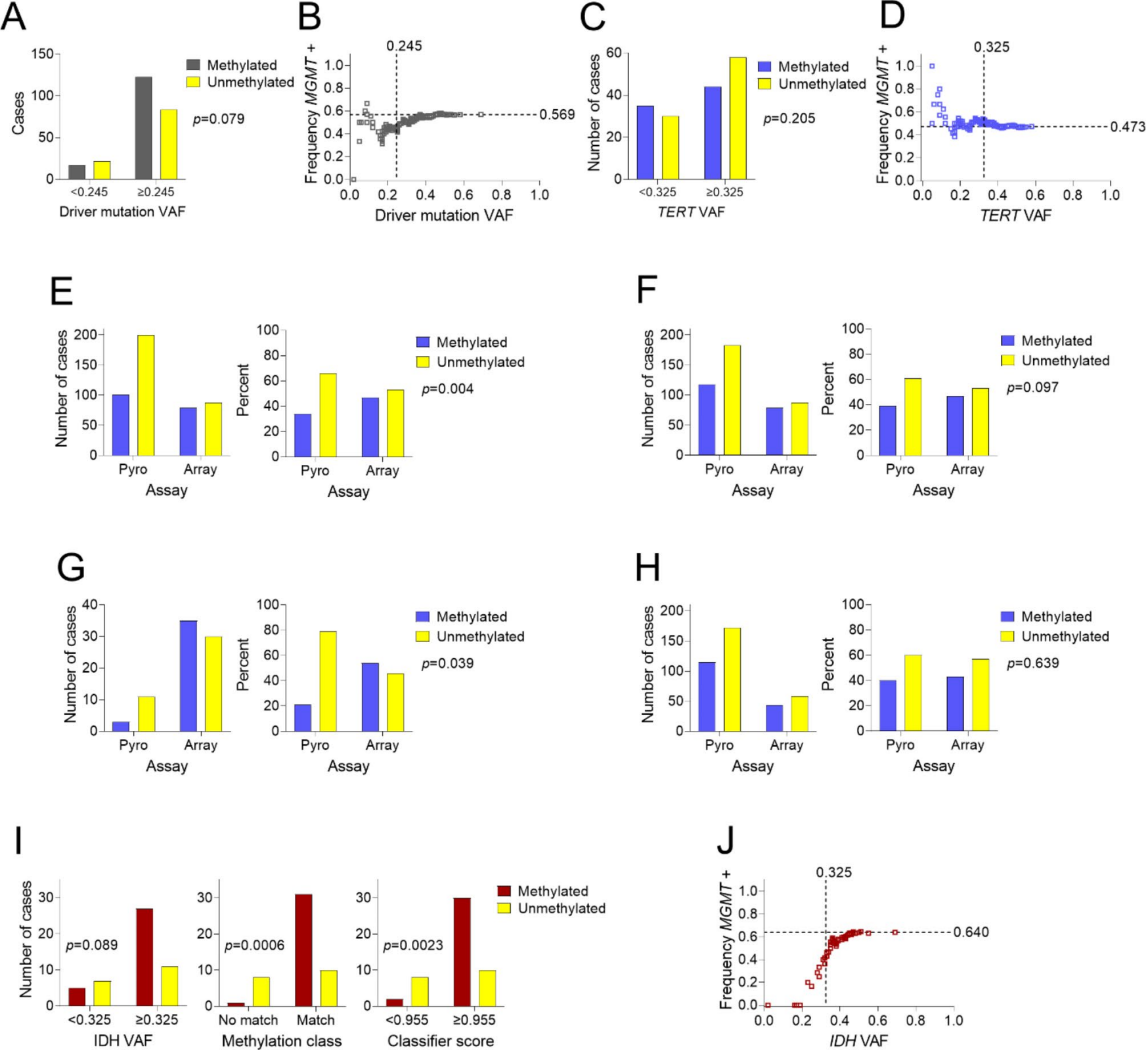
***MGMT*promoter methylation trends in DNA methylation array samples**. (**A**) Frequency of positive results for all glioma array samples above and below the cutoff of VAF = 0.245. (**B**) Cumulative mean frequency of positive results for all glioma array samples as a function of VAF. (**C**) Frequency of positive results for GBM array samples above and below the cutoff of *TERT* VAF = 0.325. (**D**) Cumulative mean frequency of positive results in GBM array samples as a function of *TERT* VAF. (**E**) Frequency of positive results in GBM pyrosequencing samples using *MGMT* cutoff of 10.0% versus GBM array samples. (**F**) Frequency of positive results in GBM pyrosequencing samples using *MGMT* cutoff of 7.28% versus GBM array samples. (**G**) Frequency of positive results in GBM pyrosequencing samples with *TERT* VAF < 0.115, using *MGMT* cutoff of 7.28%, versus GBM array samples with *TERT* VAF < 0.325. (**H**) Frequency of positive results in GBM pyrosequencing samples with *TERT* VAF ≥ 0.115, using *MGMT* cutoff of 7.28%, versus GBM array samples with *TERT* VAF ≥ 0.325. (**I**) Frequency of positive results for IDHmut astrocytoma above and below the cutoff of *IDH* VAF = 0.325 (left), by methylation class match (center), and above and below the cutoff of classifier score = 0.955 (right). (**J**) Cumulative mean frequency of positive results in IDHmut astrocytoma array samples as a function of *IDH* VAF (Fisher’s exact test for panels A, C, E, F, G, H, I; GBM: IDH-wildtype glioblastoma, IDHmut astrocytoma: IDH-mutant astrocytoma)

When pooled together, gliomas in which *MGMT* promoter methylation was determined by DNA methylation array using the Bady algorithm showed a VAF cutoff of 0.245 (Fig. 3A, Supplementary Figure S4A). The maximal proportion of cases with *MGMT* promoter methylation was 57% compared to 49% by pyrosequencing (Fig. 3B versus Fig. 2B). The shape of the curve at lower VAFs was also different for methylation array than for pyrosequencing. The likelihood of *MGMT* promoter methylation dropped to 31% at a VAF of 0.17, then surprisingly rose slightly with further decrease in VAF before dropping again at VAFs below 0.05 (Fig. 3B). Based on subset analyses, this paradoxical interval between VAFs 0.10–0.17 was driven by IDHwt GBMs, as GBM samples with low *TERT* promoter VAFs were at least as likely to be *MGMT* promoter methylation-positive as those with higher VAFs (Fig. 3 C-D, Supplementary Figure S4B). By array, 79 of 167 IDHwt GBM samples (47%) tested positive, compared to only 101 of 301 (34%) by pyrosequencing (*p* = 0.004, Fig. 3E).

Further analyses suggested that variation in *MGMT* assay results between pyrosequencing and methylation array for IDHwt GBM may be driven by differing *MGMT* pyrosequencing cutoff values and divergent results in low-VAF GBMs specifically. The original work validating the Bady algorithm compared methylation array data to pyrosequencing data with a cutoff of 7.28% CpG site methylation for a positive result [41], in contrast to the generally established pyrosequencing cutoff of 10.0%. When IDHwt GBM pyrosequencing data were adjusted downward using the 7.28% cutoff, more cases (118 of 301, 39%) tested positive, which brought pyrosequencing closer to genomic methylation array percentages (*p* = 0.097, Fig. 3F). Subgroup analysis by *TERT* VAF (also using 7.28% CpG site methylation) showed that the divergent results were most evident in low-VAF GBMs. Only 3 of 14 GBM pyrosequencing samples (21%) below the pyrosequencing-specific VAF cutoff of 0.115 tested positive, versus 35 of 65 array samples (54%) below the array-specific VAF cutoff of 0.325 (*p* = 0.039, Fig. 3G). For GBM samples with VAF at or above the respective cutoffs, 115 of 287 pyrosequencing samples (40%) tested positive, compared to 44 of 102 array samples (43%) (*p* = 0.639, Fig. 3H). These results indicate that the assays are comparable in samples with higher tumor cellularity, but in cases of low cellularity, DNA methylation array may be a more reliable measure of *MGMT* status.

In contrast to IDHwt GBMs, IDHmut astrocytomas tested by methylation array showed less frequent positive results in samples with IDH VAF < 0.325 (Supplementary Figure S4C), similar to pyrosequencing (Fig. 3I-J versus Fig. 2I-J). As expected, IDHmut astrocytomas with high scores by the methylation classifier (cutoff score = 0.955 by Cutoff Finder, Supplementary Figure S4D) were more likely to be *MGMT* promoter methylation-positive (Fig. 3I). All 29 IDHmut oligodendrogliomas analyzed by DNA methylation array tested positive for *MGMT* promoter methylation, even with VAFs as low as 0.18 (Supplementary Table S3). Outcomes of Cutoff Finder categorical Fisher’s exact tests for pyrosequencing and DNA methylation array data are summarized in Supplementary Table S5.

### Predictive value of driver mutation VAF in MGMT promoter testing and Tumor cellularity estimation

**Fig. 4:**
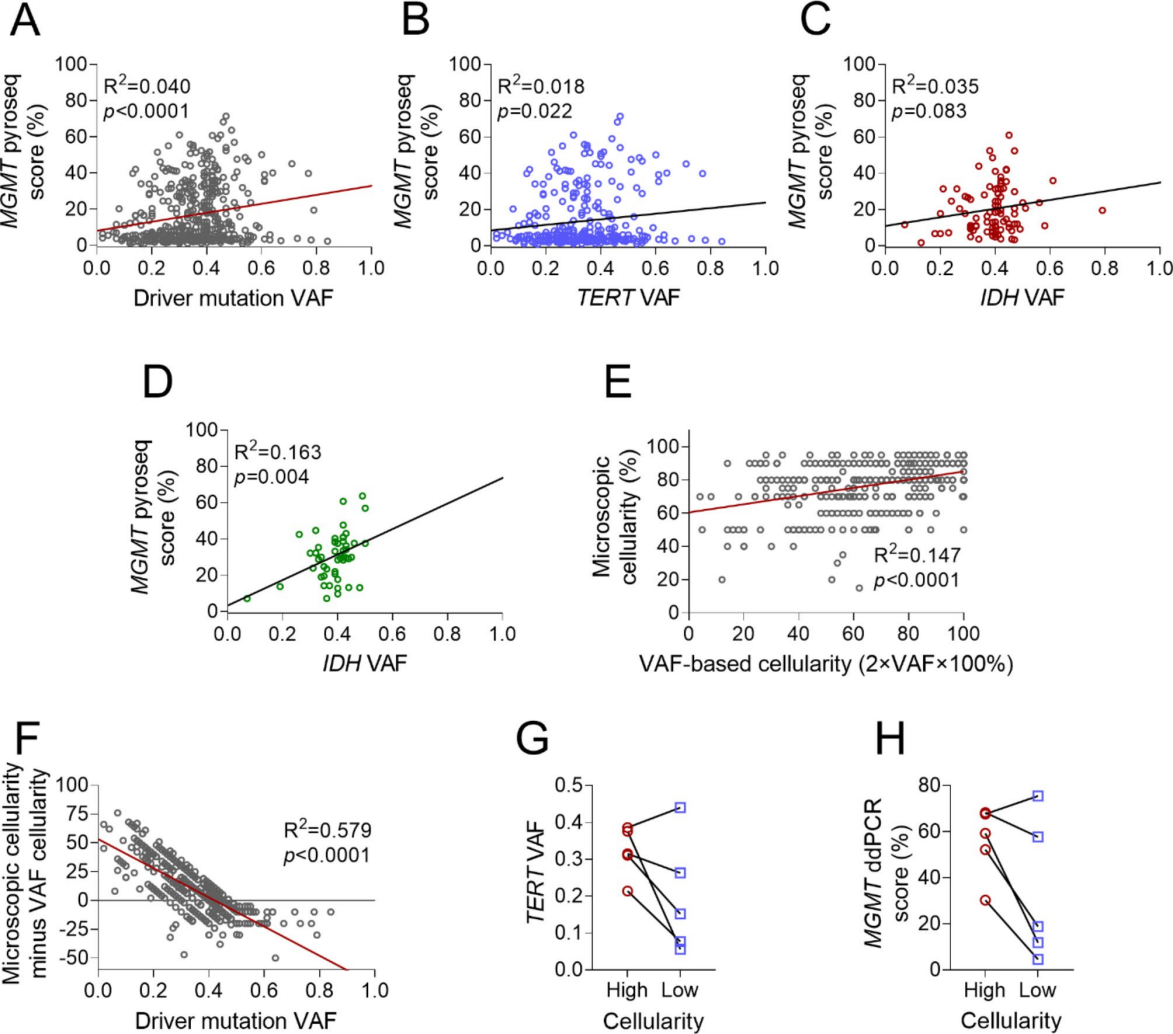
**Driver mutation VAF**, ***MGMT*promoter methylation scores, and tumor cellularity**. (**A**) Linear regression of *MGMT* promoter pyrosequencing score versus driver mutation VAF for all glioma samples. (**B**) Linear regression of *MGMT* promoter pyrosequencing score versus *TERT* promoter mutation VAF for GBM. (**C**) Linear regression of *MGMT* promoter pyrosequencing score versus *IDH* mutation VAF for IDHmut astrocytoma. (**D**) Linear regression of *MGMT* promoter pyrosequencing score versus *IDH* mutation VAF for IDHmut oligodendroglioma. (**E**) Linear regression of microscopically estimated cellularity versus cellularity calculated from driver mutation VAF (2×VAF×100%) for all glioma samples. (**F**) Differences between microscopically estimated cellularity and cellularity calculated from VAF (Y-axis) plotted as a function of VAF (X-axis), for all glioma samples. (**G**) *TERT* promoter mutation VAF by ddPCR in high versus low cellularity areas of GBM tissue samples. (**H**) *MGMT* promoter methylation score by ddPCR in high versus low cellularity areas of GBM tissue samples (GBM: IDH-wildtype glioblastoma, IDHmut astrocytoma: IDH-mutant astrocytoma, IDHmut oligodendroglioma: IDH-mutant and 1p/19q co-deleted oligodendroglioma, pyroseq: pyrosequencing, ddPCR: droplet digital PCR)

Simple linear regression showed that *MGMT* promoter methylation pyrosequencing scores positively correlated with driver mutation VAF among all gliomas (slope = 24.8, R^2^ = 0.040, *p* < 0.0001, Fig. 4A). Among the three major glioma subtypes, similar positive correlations were found for *MGMT* promoter methylation and *TERT* promoter VAF in IDHwt GBM (Fig. 4B), and for *IDH* VAF in both IDHmut astrocytoma (non-significant) and IDHmut oligodendroglioma (Fig. 4C-D). There was also a positive correlation between microscopically estimated tumor cellularity and cellularity calculated by doubling driver mutation VAF (slope = 0.246, R^2^ = 0.147, *p* < 0.0001, Fig. 4E), although there were numerous cases in which VAF did not align with microscopic estimates of tumor cellularity. To further investigate, differences between microscopy-based cellularity and VAF-based cellularity were plotted against VAF for each case. This showed a negative correlation (slope = − 126.7, R^2^ = 0.579, *p* < 0.0001, Fig. 4F), indicating that when VAF was low, microscopy-based tumor cellularity estimates tended to be higher than what was calculated by VAF.

To further assess the relationship between tumor cellularity, driver VAF, and *MGMT* assay outcomes, we performed additional analyses on 5 IDHwt GBM samples. For each sample, areas of high and low tumor cellularity were marked via light microscopy of H&E slides. *TERT* VAF and *MGMT* promoter methylation were measured in parallel by ddPCR on tissue from each area (Table 2). In 4 out of 5 cases, both *TERT* VAF (Fig. 4G) and *MGMT* promoter methylation (Fig. 4H) were lower in less cellular portions of tumors. Although the *MGMT* promoter methylation scores dropped in paucicellular areas, they remained above the ddPCR cutoff for a positive result (4.0%). In one case, *MGMT* promoter methylation increased (from 67.6 to 75.5%) in parallel with *TERT* VAF (from 0.386 to 0.440) although the microscopic estimate of cellularity fell (from 60 to 40%). This further reinforces the suggestion that driver mutation VAF may be a more objective and accurate measure of tumor purity than visually estimated cellularity.

### False negative *MGMT* promoter pyrosequencing results in cases with low driver mutation VAF

**Fig. 5:**
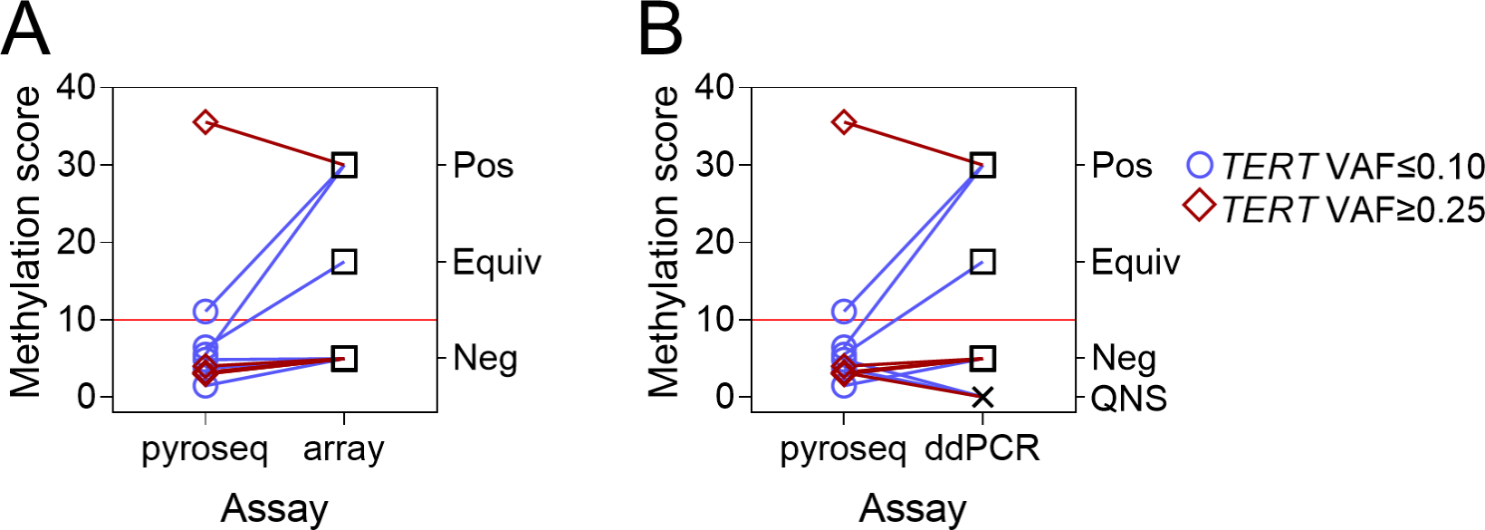
**False negative results in IDH-wildtype glioblastoma with low*TERT*VAF**. (**A**) *MGMT* promoter methylation results for 12 GBM samples (6 with *TERT* VAF ≤ 0.10, 6 with *TERT* VAF ≥ 0.25) comparing initial pyrosequencing methylation scores (left Y-axis, cutoff for positive = 10.0%, horizontal solid red line) to results on re-testing with DNA methylation array (right Y-axis). (**B**) *MGMT* promoter methylation results for the same 12 GBM samples comparing initial pyrosequencing methylation levels to results on re-testing with ddPCR. (GBM: IDH-wildtype glioblastoma, pos: positive, equiv: equivocal, neg: negative, QNS: quality/quantity of DNA not sufficient for reliable test result, ddPCR: droplet digital PCR)

When glioma driver mutation VAF is low, *MGMT* promoter methylation scores by pyrosequencing also tend to be lower, and negative test results are more frequent. Based on the divergence in *MGMT* promoter pyrosequencing outcomes with variable VAF, we investigated whether original pyrosequencing results were false-negatives by re-testing samples with DNA methylation array and ddPCR. These alternative methods were chosen based on the sensitivity of DNA methylation array in low-VAF IDHwt GBM samples (Fig. 3C-G), and on the ability of ddPCR to identify low-abundance targets [42]. A cohort of 6 IDHwt GBM samples with *TERT* VAF ≤ 0.10 (lower than VAF cutoff values identified by both Cutoff Finder and regression, Fig. 2E-H) and a sex- and age-matched control cohort with *TERT* VAF ≥ 0.25 (higher than both VAF cutoff values) were re-tested, using the same FFPE tissue blocks as initial pyrosequencing and NGS assays. Results are illustrated in Fig. 5A (pyrosequencing vs. array) and Fig. 5B (pyrosequencing vs. ddPCR) and detailed in Table 3. In the low-VAF cohort, samples from patients #2 and #3 both had *MGMT* promoter methylation status re-classified on re-testing. Sample #2 was called positive by array and equivocal by ddPCR, while the opposite held true for sample #3. Both samples had pyrosequencing promoter methylation levels near the positive cutoff, low *TERT* VAF, and favorable TMZ responses (survival of 60.3 and 20.8 months, respectively). No cases from the high-VAF cohort had their *MGMT* promoter methylation status changed on re-testing. DNA was insufficient for ddPCR in 3 cases (2 from the low-VAF cohort and 1 from the high-VAF cohort). These results demonstrate that DNA methylation array analysis and ddPCR can identify false-negative *MGMT* pyrosequencing results in low-VAF samples.

**Table 3:** Re-testing pyrosequencing samples with DNA methylation array and droplet digital PCR

Patient	*Sample	Sex	Age	*TERT* VAF	*MGMT* pyroseq result	*MGMT* array result	*MGMT* ddPCR result
#1	Pyro-311	M	61	0.02	1.49% (negative)	negative	negative
#2	Pyro-293	F	55	0.02	6.44% (negative)	equivocal	positive
#3	Pyro-049	M	53	0.04	5.52% (negative)	positive	equivocal
#4	Pyro-023	F	47	0.07	3.73% (negative	negative	QNS
#5	Pyro-081	F	69	0.10	11.06% (positive)	positive	positive
#6	Pyro-182	F	69	0.10	4.85% (negative)	negative	QNS
#7	Pyro-083	F	46	0.25	2.97% (negative)	negative	negative
#8	Pyro-200	M	53	0.28	3.18% (negative)	negative	QNS
#9	Pyro-152	F	56	0.35	4.01% (negative)	negative	negative
#10	Pyro-053	F	69	0.44	35.56% (positive)	positive	positive
#11	Pyro-417	F	69	0.49	3.20% (negative)	negative	negative
#12	Pyro-243	M	61	0.78	3.03% (negative)	negative	negative

* Original pyrosequencing sample ID in Supplementary Table S2; pyroseq: pyrosequencing; ddPCR: droplet digital PCR; QNS: quality/quantity of DNA not sufficient for reliable test result

## Discussion

*MGMT* promoter methylation testing provides vital information for adult-type diffuse glioma therapy planning [14, 43–46]. Several phase 3 trials utilizing TMZ for newly diagnosed IDHwt GBM showed that the median overall survival of patients with *MGMT* promoter-methylated IDHwt GBM was 21.2–23.2 months versus 14.0-15.3 months for patients with unmethylated IDHwt GBM [1, 14, 47, 48]. Many ongoing trials therefore use *MGMT* unmethylated status as an inclusion criterion for testing therapeutic regimens that do not involve TMZ [44]. TMZ might not even be prescribed for patients with low Karnofsky performance status scores and *MGMT*-unmethylated tumors, as the benefit-to-toxicity ratio might be too low. Conversely, *MGMT* promoter methylation is often used as an inclusion criterion for glioma trials involving TMZ-sensitizing strategies. Elderly patients, whose tolerance for TMZ is generally lower, may still receive TMZ if their IDHwt GBMs have *MGMT* promoter methylation [46, 49]. Likewise, a patient taking TMZ may continue therapy even if radiology suggests tumor progression, so long as the glioma has *MGMT* promoter methylation. Thus, accurate *MGMT* promoter methylation testing is essential for patient management.

**Fig. 6:**
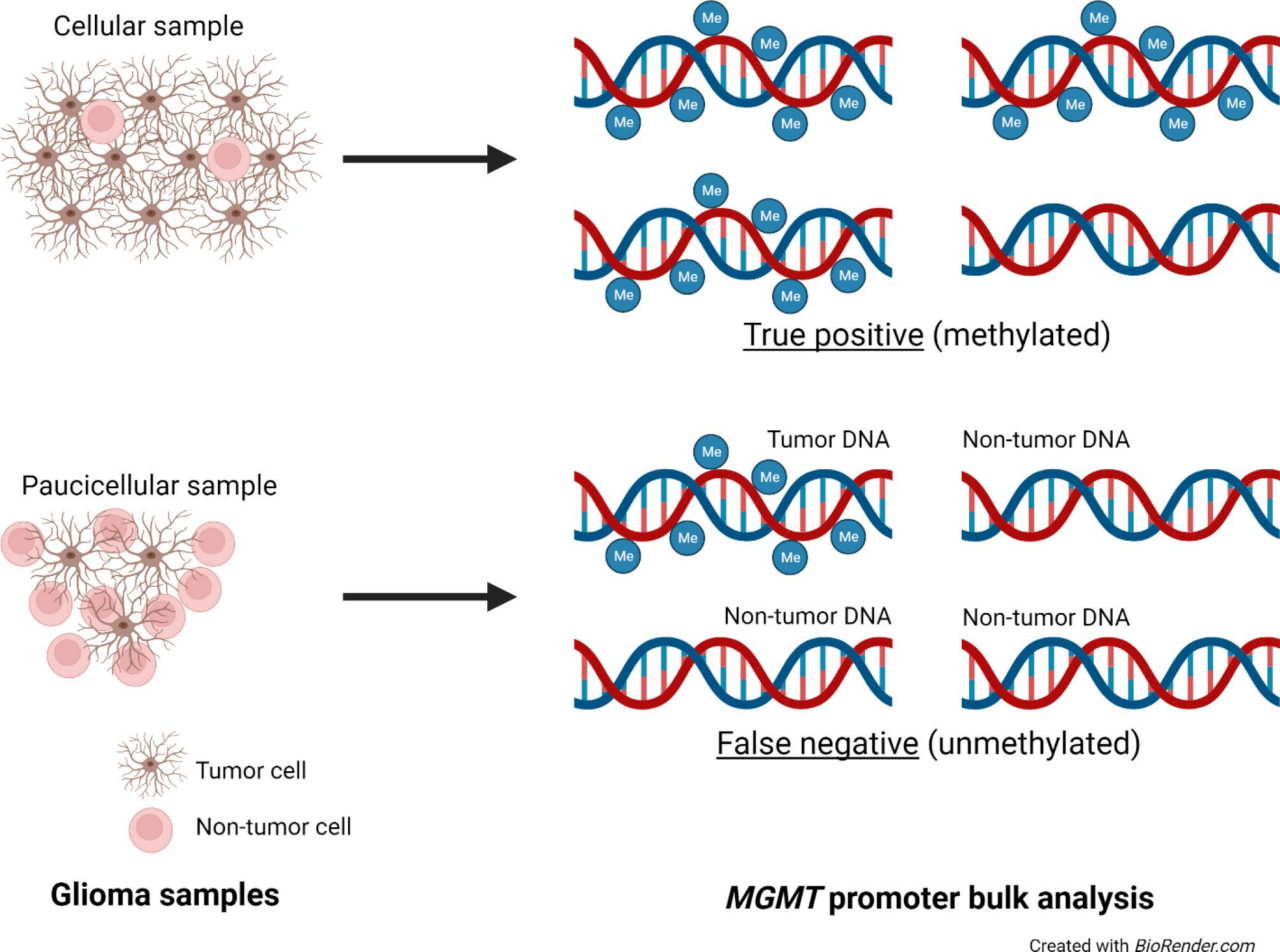
**Central hypothesis**. *MGMT* promoter methylation is pathologic, and occurs only in tumor cells. Cellular glioma samples are rich in DNA from tumor cells, whereas paucicellular glioma samples contain a large fraction of DNA from non-tumor cells, which can “dilute” positive methylation signals from tumor cell DNA, leading to false-negative results

*MGMT* promoter methylation does not occur in non-neoplastic cells, meaning they can upregulate *MGMT* gene expression and be less sensitive to TMZ than glioma cells with *MGMT* promoter methylation [50]. Since infiltrative glioma samples are always an admixture of tumor and non-tumor cells, and *MGMT* testing is done on bulk tissue samples, an excess of non-tumor cells can mask a positive signal from glioma cells (Fig. 6). Accurate results therefore depend on adequate tumor cellularity. 70% tumor cellularity is the usual benchmark for specimen acceptance, but light microscopic estimates are highly subjective. Moreover, it is unusual for specimens to be rejected for testing even if they do not reach the 70% benchmark. Thus, false-negative results are possible in some instances, although to date the frequency of such cases and objective ways of catching them have been unclear.

Here, we show that glioma driver mutation VAF, which is included in most NGS reports, can serve as a useful metric for judging the reliability of *MGMT* promoter methylation pyrosequencing results, more so than microscopic cellularity estimates. Based on these data, driver VAF should be considered when interpreting pyrosequencing results for clinical decision-making, and when considering alternative testing methods with higher sensitivity, such as ddPCR. We also show that, despite the widely used minimum cellularity of 70%, results may be considered reliable even when IDHwt GBM cellularity is much lower (35–40%). In contrast, cellularity of 60–80% appears to be needed for IDH-mutant astrocytoma and IDH-mutant oligodendroglioma. The different VAF/cellularity cutoffs for tumor types could be related to differences in intratumoral heterogeneity of *MGMT* promoter methylation in IDH-mutant versus IDH-wildtype gliomas [51, 52]. Tumors with greater heterogeneity would require higher purity to detect positive results on bulk analyses. The lower cutoff for IDHwt GBM may also be due to more genomic instability, which might cause small copy number alterations involving the *TERT* promoter locus in a subset of tumors [53]. Even so, previous work, including our own, shows that *TERT* VAF is a reliable tumor purity marker in the majority of IDHwt GBM cases [35, 36]. The low-VAF cases ultimately identified as false negatives had *MGMT* pyrosequencing levels near the positive cutoff, and patients responded well to TMZ, as would be expected. This might help explain the unexpected benefit of TMZ reported in a subset of patients whose gliomas tested negative for *MGMT* promoter methylation [14, 54]. Based on the fraction of IDHwt GBM samples in our cohort falling below the VAF/cellularity cutoffs identified by Cutoff Finder and regression (4.3% and 12.6%, respectively) and the known frequency of *MGMT* promoter methylation in IDH-wildtype GBM of ~ 35% [55], we estimate that 1.5-4.4% of IDHwt GBM *MGMT* pyrosequencing samples may have false-negative results, potentially impacting TMZ treatment decisions.

Array-based DNA methylation profiling is revolutionizing how brain tumors are diagnosed by recognizing distinctive DNA methylation “fingerprints” [27]. Among multiple advantages is its ability to interrogate *MGMT* promoter methylation status without the need for a separate test [20, 41, 56]. Our current data suggest that it is more sensitive than pyrosequencing. Our internal validation studies also support this conclusion. Validation runs of the methylation array at our institution identified 4 cases as positive which were negative by pyrosequencing. Similarly, ddPCR can detect *MGMT* promoter methylation in paucicellular tumor samples due to low background signals [42]. The fact that ddPCR successfully detected *MGMT* promoter methylation in paucicellular areas (Table 2) also demonstrates its sensitivity.

The CpG sites analyzed by each assay may be a factor in variable sensitivity. The 2 sites analyzed by the Bady algorithm from array data and by our ddPCR assay have a particularly strong association with *MGMT* transcriptional repression and TMZ sensitivity [41]. In contrast, the Qiagen™ pyrosequencing assay utilized at Northwestern interrogates 4 CpG sites in Exon 1 of the *MGMT* promoter (see methods), none of which correspond to the 2 utilized in the Bady algorithm and our ddPCR assay. CpG sites for pyrosequencing assays are often selected due to correlation with CpG methylation levels across the entire promoter region rather than their individual importance in *MGMT* transcriptional regulation [57]. More widespread use of genomic DNA methylation arrays and/or ddPCR in *MGMT* promoter methylation assessment may be beneficial in glioma diagnostic testing, due to the apparent sensitivity of both tests.

One limitation of this study is the retrospective nature of the cohort. Another is the lack of cases in which both pyrosequencing and methylation array were done. A third is the low number of IDHwt GBMs with both *MGMT* unmethylation and low VAF, which limited the statistical power of analyses of variation in positivity rates and variation in methylation level. Finally, this approach obviously cannot work in the minority of IDHwt GBMs lacking *TERT* promoter mutation.

## Conclusions

In summary, our findings demonstrate the value of driver mutation VAF as a quality assurance tool in *MGMT* promoter methylation testing of adult-type diffuse gliomas. VAF is particularly useful for identifying cases at risk of being falsely negative. This also provides another rationale for routine NGS of glioma samples (or targeted testing for driver mutations via ddPCR) along with *MGMT* testing, as mutation data not only refines the histopathologic diagnosis, but also indicates how reliable the *MGMT* result is likely to be. Future studies could focus on the recently recognized role of *MGMT* promoter methylation intratumoral heterogeneity in glioma biology and response to therapy [51, 52]. The roles of individual CpG sites within the *MGMT* promoter, and of other transcriptional regulatory elements such as the *MGMT* gene enhancer [58] could also be studied further.

### Electronic supplementary material

Below is the link to the electronic supplementary material.

Supplementary Material 1

Supplementary Material 2

Supplementary Material 3

Supplementary Material 4

## Data Availability

De-identified case data is available in Supplementary Tables S2 and S3. Additional materials are available from the corresponding author upon request.

## List of Abbreviations

ddPCR: Droplet digital polymerase chain reaction
FFPE: Formalin-fixed, paraffin-embedded
H&E: Hematoxylin & Eosin
IDH: Isocitrate dehydrogenase
IDHmut astrocytoma: IDH-mutant astrocytoma
IDHmut oligodendroglioma: Oligodendroglioma, IDH-mutant and 1p/19q co-deleted
IDHwt GBM: IDH-wildtype glioblastoma
MGMT: O^6^-methylguanine-DNA-methyltransferase
NGS: Next generation sequencing
NSTB: Nervous system tumor bank
OCAV3: Oncomine comprehensive NGS assay, version 3
QNS: Quantity/quality not sufficient
TMZ: Temozolomide
VAF: Variant allelic frequency
WHO: World health organization
