# Supplementary material for: Variant allelic frequencies of driver mutations can identify gliomas with potentially false-negative MGMT promoter methylation results
Source: Acta Neuropathol Commun. 2023 Nov 2;11:175. doi: 10.1186/s40478-023-01680-0 (PMC10623846; doi:10.1186/s40478-023-01680-0)
Supplement: Supplementary file 4 — Supplementary Material 4 [file 40478_2023_1680_MOESM4_ESM.docx]

**Supplementary information**

for

**Variant allelic frequencies of driver mutations can identify gliomas with potentially false-negative *MGMT* promoter methylation results**

Matthew McCord^1^, Pouya Jamshidi^1^, Vineeth Thirunavu^2^, Lucas Santana-Santos^1^, Erica Vormittag-Nocito^1^, David Dittman^1^, Stephanie Parker^1^, Joseph Baczkowski^1^, Lawrence Jennings^1^, Jordain Walshon^2^, Kathleen McCortney^2^, Kristyn Galbraith^3^, Hui Zhang^4^, Rimas V. Lukas^5,6^, Roger Stupp^2,5,6^, Karan Dixit^5,6^, Priya Kumthekar,^5,6^ Amy B. Heimberger,^2,6^ Matija Snuderl^3^, and Craig Horbinski^1,2,6^

^1^Northwestern University Feinberg School of Medicine, Department of Pathology

^2^Northwestern University Feinberg School of Medicine, Department of Neurological Surgery

^3^New York University Langone Health, Department of Pathology

^4^Northwestern University Feinberg School of Medicine, Department of Preventive Medicine

^5^Northwestern University Feinberg School of Medicine, Department of Neurology

^6^Lou and Jean Malnati Brain Tumor Institute of the Robert H. Lurie Comprehensive Cancer Center, Northwestern University

**SUPPLEMENTARY METHODS**

*Next generation sequencing*

For all cases, extracted DNA concentration was quantified using Qubit™ ds DNA High-Sensitive Assay kit (Thermo Fisher Scientific, Waltham, MA, USA) on the Qubit fluorometer (Thermo Fisher Scientific, Waltham, MA, USA). All library preparation for Oncomine Comprehensive version 3 NGS (Thermo Fisher Scientific, Waltham, MA, USA) was performed according to manufacturer’s instructions. Multiplex PCR amplification was conducted using a DNA concentration of approximately 20 ng as input for both DNA and RNA. For sequencing, prepared libraries were loaded according to manufacturer’s instructions onto Ion 540™ Chips (Thermo Fisher Scientific, Waltham, MA, USA) and prepared using the Ion Chef™ System. Sequencing was performed using the Ion S5™ XL Sequencer (Thermo Fisher Scientific, Waltham, MA, USA). The data was mapped to the human genome assembly 19 using TMAP aligner with default parameters. Variants were called using torrent variant caller (TVC). Variants were annotated and filtered using Ion Reporter™ Software (v. 5.6) (Thermo Fisher Scientific, Waltham, MA, USA).

PGDx next-generation sequencing Elio™ tissue complete assay was performed according to the manufacturer's recommendations. Briefly, genomic DNA extracted from formalin-fixed paraffin-embedded tissue was quantified using a fluorometer. DNA molecules were mechanically sheared to a target size of 200 bp and subjected to a magnetic bead purification step to remove smaller fragments. Fragmented DNA was end-repaired, phosphorylated, and adenylated. Indexed adapters were then ligated to the A-tailed DNA molecules. Unincorporated adapters and reagents were removed by magnetic bead purification. Adapter-ligated DNA was enriched by PCR amplification. Primer dimers and residual reagents were removed by magnetic bead purification. Library quality was assessed using a DNA fragment analyzer prior to hybrid capture. The adapter-ligated library was hybridized with biotinylated RNA library baits, and targeted regions were captured using magnetic streptavidin coated beads. Captured libraries were purified to remove baits and incompletely hybridized DNA fragments. Captured libraries were enriched by PCR amplification. Primer dimers and residual reagents were removed by magnetic bead purification. Final library quality was assessed using a DNA fragment analyzer prior to sequencing. Sample libraries were quantified and normalized into a sequencing pool of up to 15 samples and the external control. Pooled sample libraries were fluorometrically quantified, loaded on a sequencing flow cell and sequenced using a NextSeq® 550Dx instrument which was pre-qualified by PGDx. Sequence data was processed using the PGDx elio platform software. The software contains a user interface that tracks sample status from sequencing through analysis and reporting. Users configure sequencing runs, and an automated pipeline of software for bioinformatic analysis identifies and reports genomic alterations. PGDx is an FDA-cleared medical device and uses a proprietary bioinformatics pipeline. First, the pipeline generated FASTQ files containing sequences and quality scores for each sample from Illumina BCL files. The FASTQ files were then aligned to a reference genome (GCRh37) to generate BAM files, which were processed for variant calling of different alteration types (SNVs, indels, amplifications, translocations, and MSI). SNVs and indels were then used to determine TMB scores reported as mutations per megabase.

**SUPPLEMENTARY FIGURES**


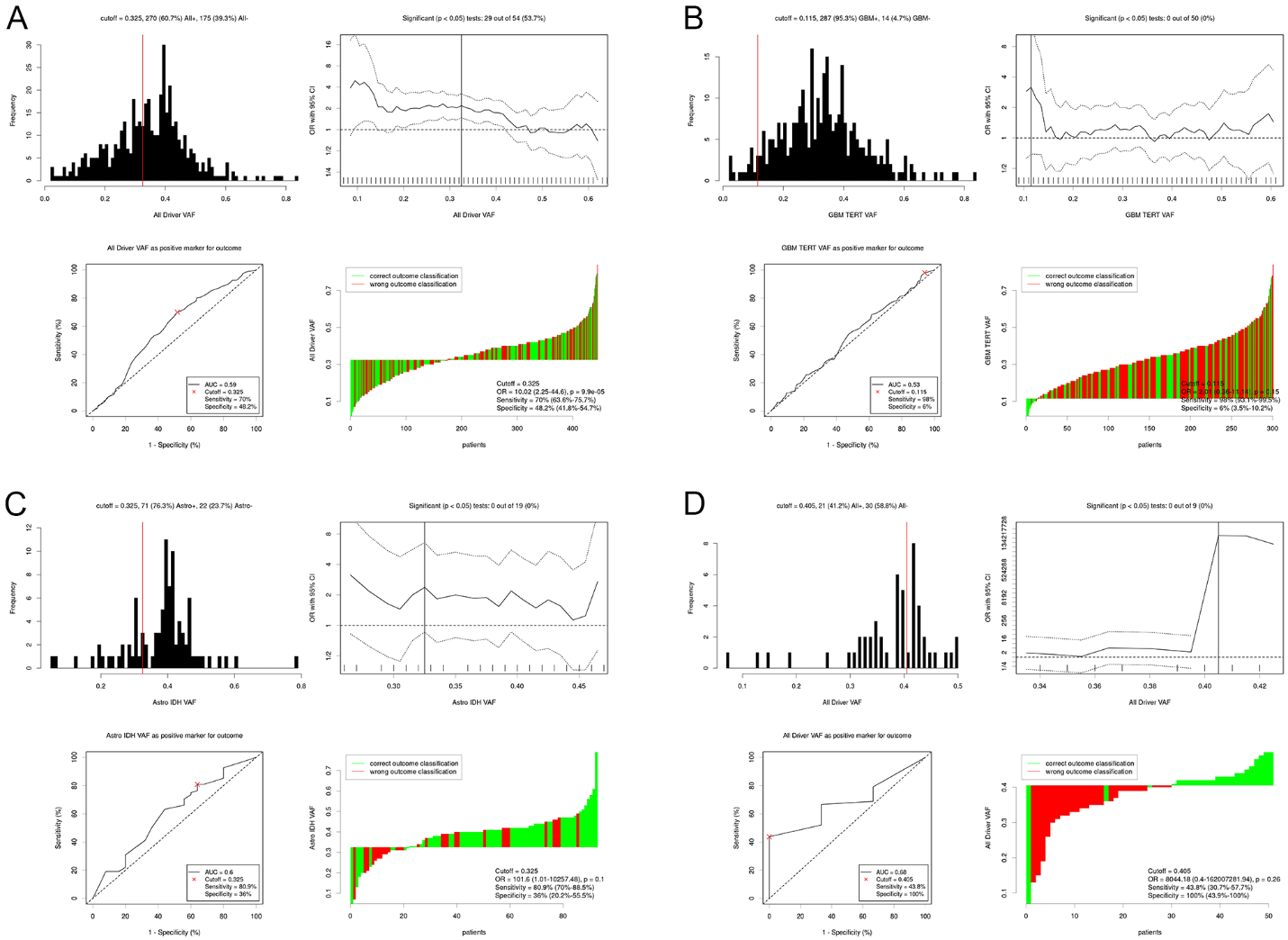


**Supplementary Figure S1: Cutoff Finder results for pyrosequencing data.** (A) All glioma samples combined. (B) IDH-wildtype glioblastoma. (C) IDH-mutant astrocytoma. (D) IDH-mutant and 1p/19q co-deleted oligodendroglioma.


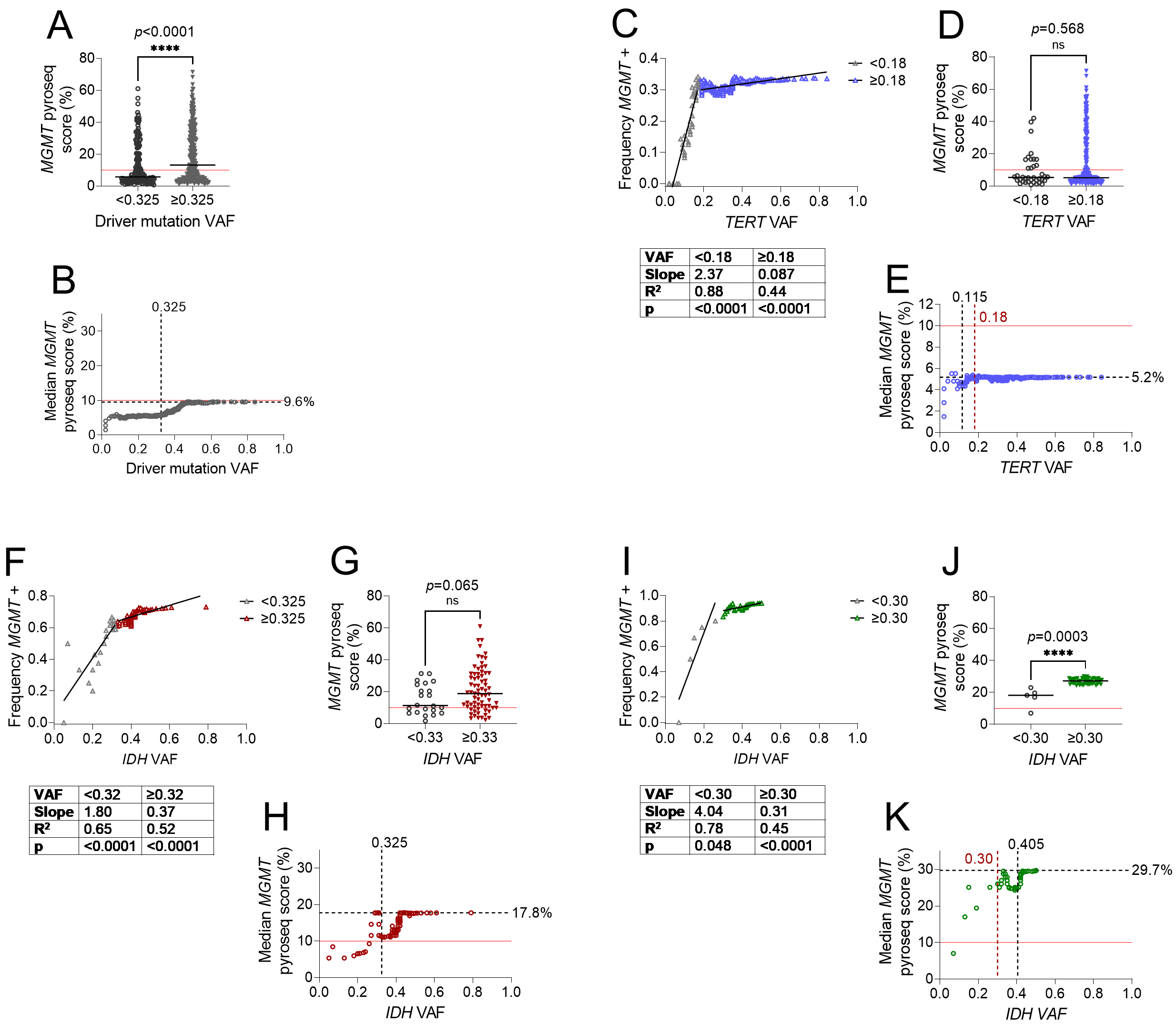


**Supplementary Figure S2: Additional analysis of outcome distribution for *MGMT* pyrosequencing.** (A) Median *MGMT* promoter pyrosequencing scores for all gliomas above and below the cutoff value of VAF=0.325. (B) Trends in cumulative median *MGMT* promoter methylation level with increasing VAF for all gliomas. (C) Results of two-part linear regression to identify inflection points and optimal *TERT* VAF cutoff for the data curve shown in Figure 2F, showing cumulative mean frequency of positive *MGMT* pyrosequencing results in IDH-wildtype glioblastoma. (D) Median *MGMT* pyrosequencing scores above and below the cutoff value of *TERT* VAF=0.18 for IDH-wildtype glioblastoma. (E) Trends in cumulative median *MGMT* pyrosequencing scores with increasing *TERT* VAF for IDH-wildtype glioblastoma. Similar results are shown for IDH-mutant astrocytoma (F-H) and for IDH-mutant and 1p/19q co-deleted oligodendroglioma (I-K). (Horizonal dashed black lines: median values for cohort; Horizontal solid red lines: *MGMT* positivity cutoff of 10%; Vertical dashed black lines: cutoff values identified by Cutoff Finder; Vertical dashed red lines: cutoff values identified by multi-part linear regression Pyroseq: pyrosequencing; *****p*<0.0001).


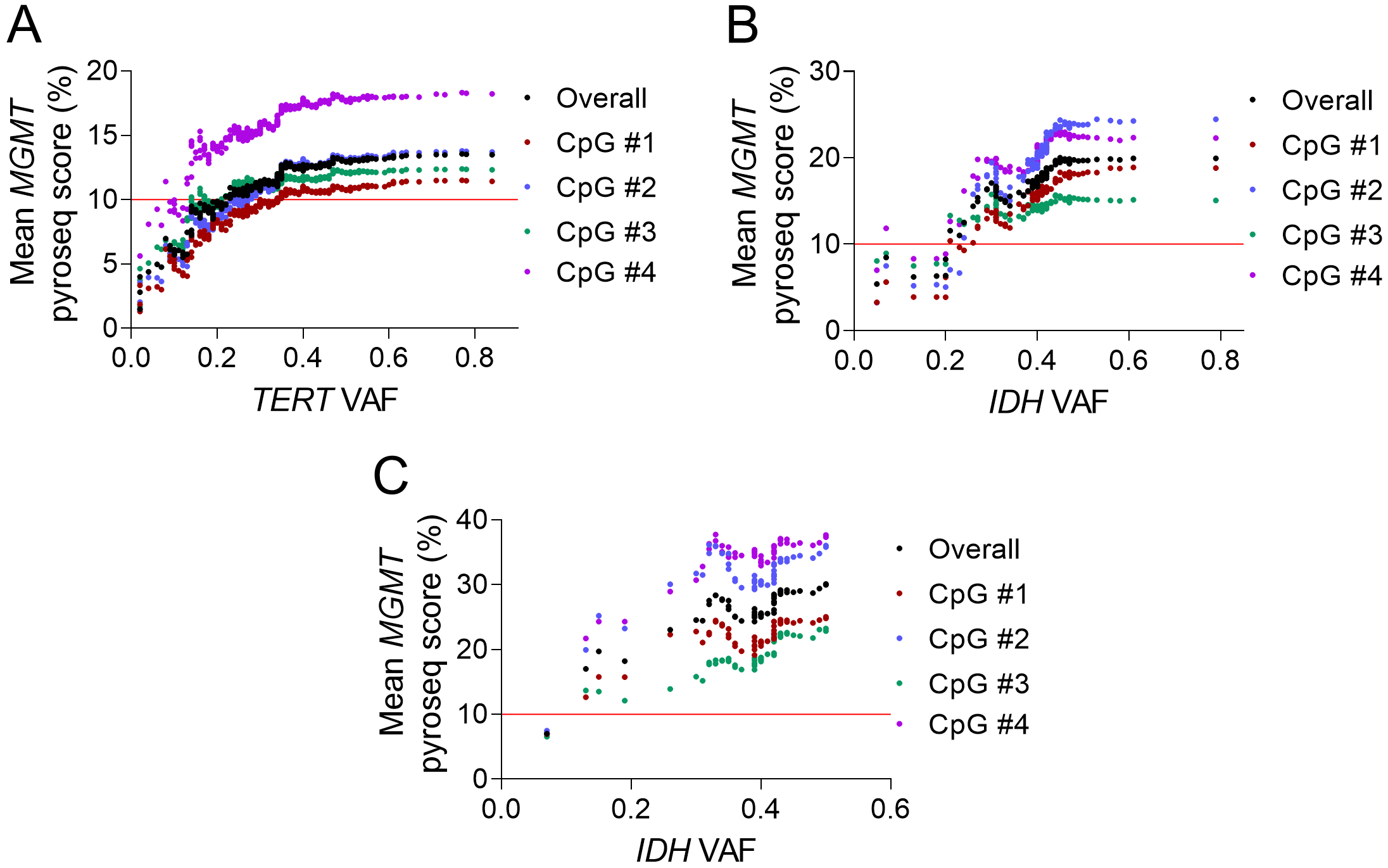


**Supplementary Figure S3: Trends in *MGMT* pyrosequencing scores for individual CpG sites:** Trends in cumulative mean *MGMT* methylation score for the 4 individual CpG sites measured by pyrosequencing, along with the overall score (mean of the 4 sites) for each sample, with increasing VAF. Trends are shown for IDHwt GBM (A), IDH-mut astrocytoma (B) and IDH-mut oligodendroglioma (C). Horizontal solid red lines: *MGMT* positivity cutoff of 10%. Pyroseq: pyrosequencing.

**
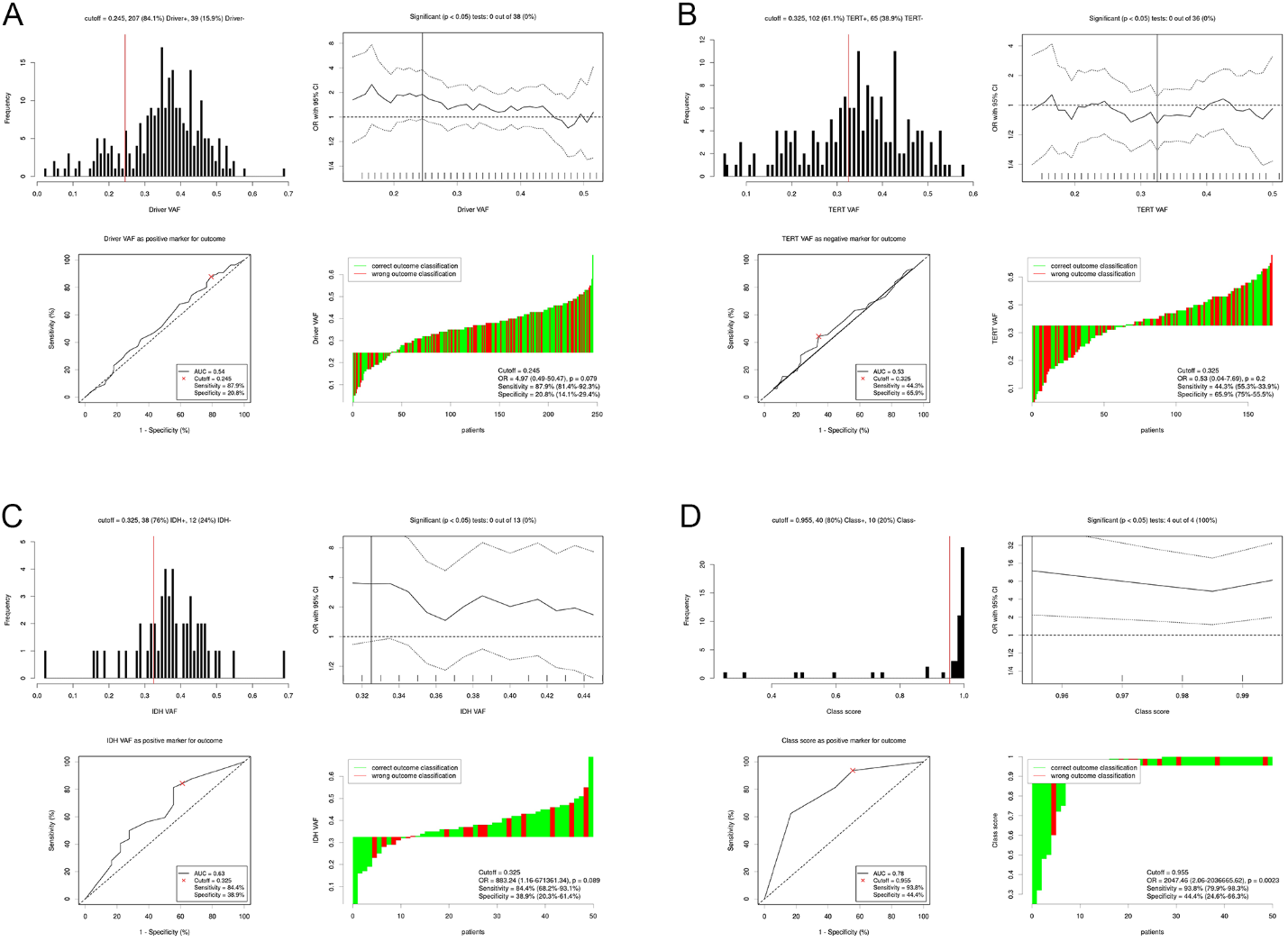
**

**Supplementary Figure S4: Cutoff Finder results for DNA methylation array data.** (A) All glioma samples combined. (B) IDH-wildtype glioblastoma. (C) IDH-mutant astrocytoma, VAF analysis. (D) IDH-mutant astrocytoma, methylation classifier score analysis.

**SUPPLEMENTARY TABLES**

**Supplementary Table S1: Sequences and fluorophores for *MGMT* promoter assessment by droplet digital PCR.**

| ***MGMT*-methylated (positive)** | |
| --- | --- |
| Forward | 5’-TGTGTTTTGGATATGTTGGGATAGT-3’ |
| Reverse | 5’-AACTCCACACTCTTCCAAAAACAA-3’ |
| Probe | 5’-TTTTTGTGGTGTGTATTGTT-3’ |
| Fluorophore | Hexacholorfluorescein (HEX) |
| ***MGMT*-unmethylated (negative)** | |
| Forward | 5’-GCGTTTCGACGTTCGTAGGT-3’ |
| Reverse | 5’-CACTCTTCCGAAAACGAAACG-3’ |
| Probe | 5’-CGCAAACGATACGCACCGCGA-3’ |
| Fluorophore | Fluorescein (FAM) |

**Supplementary Table S5: Summary of data from Cutoff Finder Fisher’s exact tests**

| **Assay** | **Tumor type** | **VAF cutoff** | **Methylated/unmethylated below cutoff** | **Methylated/unmethylated above cutoff** | ***p*-value** |
| --- | --- | --- | --- | --- | --- |
| Pyro | All | 0.325 | 65/110 | 152/118 | <0.0001 |
|  | IDHwt GBM | 0.115 | 2/12 | 99/188 | 0.152 |
|  | IDH mut astro | 0.325 | 13/9 | 55/16 | 0.104 |
|  | IDHmut oligo | 0.405 | 27/3 | 21/0 | 0.259 |
| Array | All | 0.245 | 17/22 | 123/84 | 0.079 |
|  | IDHwt GBM | 0.325 | 35/30 | 44/58 | 0.205 |
|  | IDHmut astro | 0.325 | 5/7 | 27/11 | 0.089 |
|  | IDHmut oligo* | N/A | N/A | N/A | N/A |

VAF: variant allelic frequency; IDHwt GBM: glioblastoma, IDH-wildtype; IDHmut astro: astrocytoma, IDH-mutant; IDHmut oligo: oligodendroglioma, IDH-mutant and 1p/19q co-deleted; *all IDHmut oligo samples tested by array were resulted as “methylated”
